# Editorial: Advances in nano-scale systems with optics (nano-chemical, nanomaterial, and nano-biomedicine)

**DOI:** 10.3389/fchem.2022.989153

**Published:** 2022-08-12

**Authors:** Si-Wei Zhang, Honghui He, Chao He, Martin Booth

**Affiliations:** ^1^ Department of Chemistry, Hong Kong Branch of Chinese National Engineering Research Center for Tissue Restoration and Reconstruction, The Hong Kong University of Science and Technology, Kowloon, Hong Kong SAR, China; ^2^ Shenzhen Key Laboratory for Minimal Invasive Medical Technologies, Guangdong Research Center of Polarization Imaging and Measurement Engineering Technology, Tsinghua Shenzhen International Graduate School, Tsinghua University, Shenzhen, China; ^3^ Department of Engineering Science, University of Oxford, Oxford, United Kingdom

**Keywords:** microscopic imaging technology, organic light-emitting devices (OELDs), sensor, raman spectoscopy, weak measurement technology, super-resolution microscopy

Optical properties of materials are important aspects of various recent advances in nanoscience, contributing to plenty of applications from fundamental research to industry. The cross-disciplinary design, synthesis, characterization, and applications the nano-systems are at the heart of various research directions. Novel nano-materials such as nanofibers, quantum dots, and nanoclusters have been adopted in applications such as photoelectric devices, microscopy, and optical communications. By combining organic and inorganic materials, increasingly nanoscale devices have been utilized in various integrated systems such as optical sensors, detectors, and switches, with low-power consumption, high speed, and high stability. Furthermore, optical properties of nanofiber structures inside the tissues also have been harnessed to assist clinical applications.

This special issue collects 14 excellent papers: 10 original research papers and 4 review articles, spanning microscopic imaging technology, super-resolution microscopy, organic light-emitting devices (OLED) and LED, weak measurement technology, Raman spectroscopy, and sensors.

Microscopic imaging technology (MIT) Accelerates the pace of exploring the micro world**.** Wang et al. reviewed the development of MIT and its application in micro-and nano fields (Wang et al.). Wu et al. developed an imaging technique by circular dichroism second-harmonic generation (CD-SHG) to characterize the 3D distribution of potassium titanyl phosphate (KTP) nanocrystal, which insights into nanoscale morphology of KTP and benefits the experimental configuration optimization of CD-SHG microscopy (Wu et al.). With machine learning, Zhu et al. designed a U-Net-based neural network to accelerate DNA-PAINT imaging from a widefield fluorescence image and a sparse single-molecule localization image. This approach only requires one-tenth of the original raw data but permits fast imaging and super-resolution reconstruction of microtubules as well as analyzing other SMLM datasets (Zhu et al.). Shao et al. analyzed the influence of imaging resolution on polarization properties of scattering media obtained from a Mueller matrix, which provides a criterion to decide what kind of structural information can be accurately and rapidly obtained using transmission Mueller matrix microscope with low NA objectives to assist pathological diagnosis and other applications (Shao et al.).

Super-resolution microscopy (SRM) has become a powerful tool for visualizing biological activities in both fixed and living cells. Wang et al. reviewed the development of microscope technology, summarized the properties of numerous microscopes, and discussed their applications in micro and nanotechnology (Wang et al.). Jing et al. summarized recent technical advancements in SRM, discussed together with the spectroscopic and chemical demands of the fluorophores, and highlighted some inherent challenges faced in this emerging field (Jing et al.). Sun et al. introduced a novel approach, which combines second-harmonic generation with two-photon excited fluorescence to visualize the dynamics of transdermal collagen absorption *in vivo*, providing a reliable measurement for real-time evaluation of collagen absorption and treatment effects *in vivo* (Sun et al.).

OLEDs and LEDs Liu et al. designed and synthesized a novel 3 + 2+1 coordinated iridium (III) complex for high efficient deep-blue phosphorescent OLED (Liu et al.). Chen et al. reported a promising luminescent phosphor for next-generation illuminant based core/shell CdZnSeS/ZnSeS quantum dots (QDs) (Chen et al.). QDs are promising for next-generation lighting and display. Zhang et al. emphasized the significance of detection of nanoparticles synthesized by a gas-phase method, reviewed the development of detection technology, and prospected its future (Zhang et al.).

Weak measurement technology has great potential in biomolecular detection in the frequency domain field. Xu et al. elucidated the difference between the weak measurement method and the classical measurement process and highlighted the transition conditions of the weak value enhancement. Furthermore, a transition mode of the weak and classical measurements is proposed and an optimized fitting model of the measurement results is found by performing a systematic analysis, suggesting the wide implementation of weak measurement-based detection technology (Xu et al.).

Raman spectroscopy is an essential non-destructive testing method. Li et al. investigated cold cataract cellular mechanisms in young lenses of wild-type C57BL/6J (B6WT) mice treated at different temperatures. Raman spectroscopy fluctuation reveals new mechanistic information about cold cataract formation, which is associated with the uneven distribution of lens proteins and water across lens fiber cells. Raman spectroscopy partly reveals cold temperature-induced redistribution of lens proteins such as intermediate filaments in inner fiber cells (Li et al.).

Sensors are the central part of detection. Zeng et al. developed a high-sensitive surface plasmon resonance imaging (SPRi) biosensor based on the dual-wavelength differential method. The new technique achieved a refractive index resolution of 2.24 × 10^−6^ RIU and high-throughput real-time detection biomolecular binding, which is expected to promote the development of faster and more accurate SPRi technologies (Zeng et al.). Shen et al. proposed a self-referencing interference sensor based on coherence multiplexing, which can address temperature and non-specific binding. The temperature fluctuations and specific binding experiments of protein A to IgG demonstrate that the proposed sensor can eliminate non-specific binding and temperature disturbances in real-time biomolecule detection, achieving higher detection robustness (Shen et al.).

